# Factors Influencing Lesion Formation During Radiofrequency Catheter Ablation

**Published:** 2003-07-01

**Authors:** Olaf J Eick

**Affiliations:** Sr. Scientist, Bakken Research Center Maastricht, the Netherlands

## Abstract

In radiofrequency (RF) ablation, the heating of cardiac tissue is mainly resistive. RF current heats cardiac tissue and in turn the catheter electrode is being heated. Consequently, the catheter tip temperature is always lower - or ideally equal - than the superficial tissue temperature. The lesion size is influenced by many parameters such as delivered RF power, electrode length, electrode orientation, blood flow and tissue contact. This review describes the influence of these different parameters on lesion formation and provides recommendations for different catheter types on selectable parameters such as target temperatures, power limits and RF durations.

## Introduction

### Lesion Generation due to Radiofrequency Energy Application - theoretical considerations

In RF ablation, the heating of cardiac tissue is mainly resistive. RF current is applied to the tissue via a metal electrode at the tip of the catheter, with a large skin electrode serving as indifferent electrode. The current density patterns in the tissue are determined by electrode size and geometry, electrode contact and local tissue properties. Also, of course, the current density will be proportional to the current (I) delivered by the RF generator, which, for constant resistance (R) of the electrode-tissue volume conductor is proportional to the square root of the RF power (P = I^2^ R).

Current flow through a resistive medium causes heating, which is proportional to the square of the current or, locally, to the square of the local current density. The temperature increase will locally be proportional to the energy applied per second (local power), and increases proportional to the heat capacity of the local medium. In addition, when temperature differences between adjacent areas develop because of differences in local current density or local heat capacity, heat will conduct from "hotter" to "colder" areas, causing the temperature of the former to decrease and that of the latter to increase. Additionally, heat loss to the blood pool at the surface and to intramyocardial vessels determine the temperature profile within the tissue.

The heating occurs especially in the proximity of the active electrode due to its relatively small surface area causing locally high current density as compared to the site of the indifferent electrode. Typically, living tissue will be permanently destroyed at temperatures of approximately 45° to 50° C sustained for several seconds [1].

The tissue surface is cooled by the blood flow and thus the highest temperature during radiofrequency delivery occurs slightly below the surface.

## Parameters Determining Lesion Formation

### Impact of target temperature - In Vitro Study

The impact of the target temperature on lesion size was evaluated by the author in an in vitro study. A 4-mm tip catheter (RF Marinr, Medtronic EPSystems, Minneapolis, MN) was positioned in a parallel orientation to porcine epicardium either with 0.5 N or 1.0 N contact force. A total of 48 lesions was produced with different target temperatures of 50, 60, 70 and 80° C (Atakr II, Medtronic, 100 W max. output, 60 s duration). Each setting was repeated 6 times and the average values were used for evaluation. The results are given in Table 1.

With increasing target temperature the delivered power, tip temperature and lesion dimension increased. Lesions created with a target temperature of 50° C were very small and nearly unrecognizable.

These results indicate that the lesion size could be well predicted by measuring tip temperature. However, the in vitro experiments were performed under stable flow conditions. In the in vivo situation, the flow is dependent on the ablation site and also varying over the heart cycle. These cooling effects have a strong impact on the catheter tip temperature and thus on the delivered power and also on lesion size.

### Impact of Flow - In Vitro Study

Petersen et al. evaluated the impact of convective cooling on lesion dimension [2]. In vitro strips of porcine left ventricular myocardium during different levels of convective cooling were ablated. All applications were performed with a 4-mm tip catheter in parallel orientation to the tissue and automatic power adjustment (Atakr, 75W) to reach and maintain a target temperature of 80° C for 60 seconds. The results are shown in Table 2.

With increasing flow, i.e. increasing convective cooling, lesion depth, width and volume increased due to increasing power consumption to reach and maintain target temperature. Note, that the tip temperature was not different and is thus a poor indicator for lesion size if the flow condition is not stable.

### Impact of Flow - In Vivo Study

Petersen et al. induced a total of 13 lesions in 6 pigs either at the left ventricular apex or at the mid-septum of the left ventricle [3]. A 4-mm tip catheter was used for RF delivery with a target temperature of 80° for 60 s (Atakr, 50 W maximum output). The results are shown in Table 3.

Lesions induced at the apical site were significantly smaller than those at the septal site. The blood flow is considered to be higher at the septum as compared to the apex, thus more power is consumed to maintain target temperature at the septum resulting in larger lesions. Again the tip temperature is a poor (or even contradicting) parameter for lesion size. Comparing the lesion volumes of the in vivo study with those of the in vitro study, the flow at the apex might be between 0.0 m/s and 0.1 m/s whereas the flow at the septum might me be more in the order of 0.2 m/s or higher.

### Catheter Orientation - In Vitro Study

Using the same experimental setup Petersen et al. investigated the impact of electrode orientation on lesion size [4]. A 4-mm tip catheter was used for ablation with a target temperature of 80° C for 60 s either in a parallel or perpendicular orientation on porcine tissue stripes. The contact pressure between electrode tip and tissue was 10g and the flow 0.1m/s. The results are shown in Table 4.

Perpendicular electrode orientation yielded larger lesion volume using less power than parallel electrode orientation. The target temperature was approached in all applications with a mean electrode tip temperature of 75±2° C.

Changing the 4-mm tip electrode orientation from parallel to perpendicular decreases the proportion of the electrode tip area that is in contact with the tissue and increases the proportion of the tip area that is exposed to the convective cooling of the surrounding fluid. The larger lesion volume in the perpendicular electrode orientation suggests that cooling by flow around the electrode has greater impact than contact area. However, one would expect a significantly higher power delivery in the group with larger lesion volume. This can be explained by the fact, that only a minor fraction of the energy delivered by the generator is used for the lesion production itself. [In a first approximation the power required to produce a lesion with a certain volume can be calculated as follows: Power = 1/t * lesion volume * density * specific heat * ∆T = 1/60 * 0.25 * 1.05 * 3.72 * (80-37) = 0.7 W. That means that only 0.7 W are required to produce a lesion of 250 mm3 with a target temperature of 80°C for 60 s ! Assumption: Tissue Density = 1.05g/cm3, specific heat = 3.72 J/(g*°C), t = 60 seconds, ∆T = target temperature - body temperature = 43°C.] The major part is dissipated as electrical heating of the intracavitary blood, convective heat losses from electrode to blood, electrical heating of tissue outside the lesion volume and in electrical resistance of catheter and skin and fat layers at the indifferent electrode.

### Catheter Orientation - In Vivo Study

Chan et al. published results based on in vivo data that are in conflict with those described by Petersen [5] and a likely explanation will be offered at the end of this paragraph.

In 26 dogs 144 lesions were created either in a parallel or perpendicular orientation in the right atrium with a target temperature of 75° C for 60 s using different tip lengths. The orientation was confirmed by fluoroscopy and intravascular ultrasound. For reasons of comparison only the results for the 4-mm tip catheter are given in Table 5.

The lesion volume was larger for the parallel orientation as compared to the perpendicular orientation which is in contradiction with the results from the in vitro study performed by Petersen et al.. However, all lesions were markedly smaller than in the previously summarized studies mainly due to the small lesion depth. The lesions were produced in the right atrium and the atrial wall is rather thin. The lesions would have been deeper in thicker tissue-note that 10 out of 14 for the perpendicular orientation and 9 out of 14 for the parallel orientation were transmural.

Chugh et al. who performed similar experiments in the left ventricle confirmed this [6]. They analysed 103 lesions in 20 dogs produced in the left ventricle also with a target temperature of 70° C for 60 s using different tip lengths. Table 6 summarizes the results for the 4-mm long catheter tip.

The lesion depth was markedly higher than that of the atrial applications and similar to the values that Petersen et al. reported. Although there was a trend that the lesions produced in the parallel orientation were larger the difference did not reach statistical significance.

Based on these studies one may conclude that lesion depth is only little affected by catheter tip orientation using 4-mm long tip catheters but that lesions are slightly longer in the parallel orientation as compared to the perpendicular orientation.

### Impact of Electrode Tip length

Petersen et al. evaluated the impact of electrode tip length on lesion size in 34 pigs [2]. Lesions were produced in the left ventricle in the posterior mid-septum, the left anterior free wall and in the apex using 2, 4, 6, 8, 10 and 12-mm long catheter tips. A target temperature of 80° C for 60 s was chosen with a maximum available power output of 75 W (Atakr II). The results are given in Table 7.

The lesion volume increased with increasing tip length for tip lengths between 2 and 10-mm. The lesion volume produced with an 8-mm long tip was about twice as big than that with a 10-mm tip catheter and even 3 times as big than that produced with a 4-mm tip catheter. Further increase in tip length did not result in further increase in lesion volume. With a very long catheter tip a large part is exposed to the blood flow and more energy is dissipated into the blood stream. Note that the average temperature decreased with increasing tip length and was thus a poor (or no) indicator for lesion volume. It is the amount of power that is effectively delivered to the tissue that determines lesion size. In addition, the depth did not differ between 4 and 8-mm tip catheters, the produced lesions are only wider but not necessarily deeper. Also, the applied average power was "only" 49 W for lesions created with an 8-mm tip catheter. Limiting the wattage to 50 W may reduce the likelihood of coagulum formation without compromising lesion size.

Langberg et al., who also produced lesions in the left ventricle using either 4-mm, 8-mm or 12-mm tip catheters, confirmed these results in part [7]. The target temperature was also 80° C for 60 s with a maximum available power of 100 W. The results are given in Table 8.

The power required to achieve a steady state temperature of 80° C was directly proportional to electrode size. The lesions produced by the 8-mm tip electrode were nearly twice as deep and four times as large as those made with a conventional 4-mm tip electrode. Lesions produced by the 12-mm tip electrode were intermediate in size and sometimes associated with charring and crater formation. Langberg et al. stated furthermore that ablations with larger tip electrodes caused a drop in arterial pressure and more ventricular ectopy than those with a 4-mm tip electrode.

The main difference to the results published by Petersen et al. is that lesions produced with the 8-mm tip catheter were also much deeper than those produced with the 4-mm tip catheter in the Langberg study. In concordance to the Petersen study, the use of a very large electrode did not further increase lesion size, and the tip temperature was even negatively correlated with lesion size.

 The clinical relevance of the fact that 8-mm tip catheters produce larger lesions was demonstrated by Tsai et al. [8]. In a prospective, randomized study they compared 4-mm with 8-mm tip electrodes for linear ablation of typical atrial flutter in 104 patients. They reported that the 8-mm electrode catheter achieved higher complete isthmus block rate (92% vs. 67%, p<0.05) with fewer pulses (2±1 vs. 3±1, p<0.05), shorter procedure time (24±15 vs. 31±12 minutes, p<0.05), and shorter fluoroscopy time (14±10 vs. 23±15 minutes, p<0.05).

### Impact of RF Duration

Simmers et al. evaluated the relation between RF duration and lesion size and published their results in 1994 [9]. In 11 dogs a total of 46 lesions was produced, 31 at left ventricular sites and 15 at right ventricular sites. They applied a constant power of 25 W for either 5, 10, 20, 30 and 60 seconds using a 7F, 4-mm tip electrode catheter (Mansfield-Webster, Watertown, MA, USA). The results are given in Table 9.

These results indicate that the lesion is predominantly generated within the first 10 seconds of energy delivery and reaches a maximum after 30 s. Further extension of RF delivery during power controlled RF delivery does not seem to further increase lesion size.

### Impact of Electrode-Tissue Contact - In Vitro Study

It is difficult to evaluate the impact of electrode-tissue contact in the in vivo situation since the contact pressure between electrode and tissue cannot be assessed by fluoroscopy and a direct indicator for tissue contact is lacking at present. Some studies have been published where the influence of the electrode-tissue contact has been investigated in a well controllable in vitro environment. However, the conclusions out of these studies need to be drawn carefully to avoid misleading interpretations.

**Figure 1:**
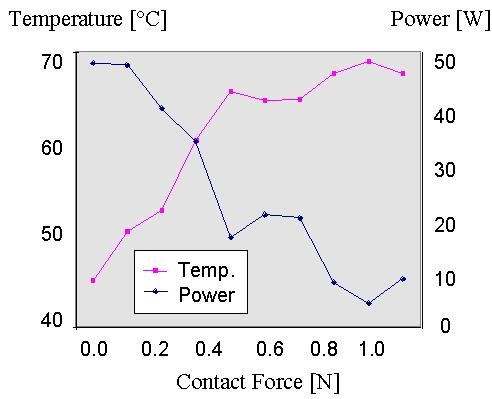
Graphical representation of the average catheter tip temperature and power for different contact forces during temperature controlled radiofrequency delivery for 30 s with a target temperature of 70° C and a maximal power of 50 W.

**Figure 2:**
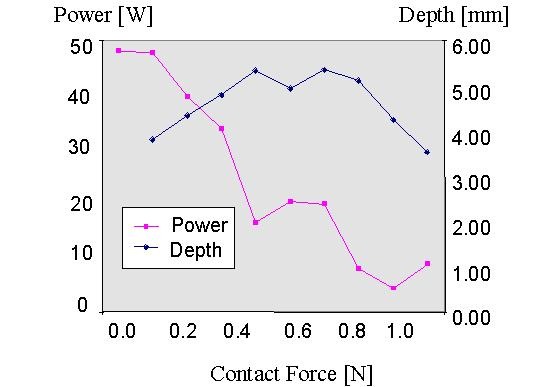
Graphical representation of the average power and the resulting lesion depth for different contact forces during temperature controlled radiofrequency delivery for 30 s with a target temperature of 70° C and a maximal power of 50 W.

The author produced in vitro ablations on porcine myocardium with a 7F, 4-mm tip electrode with different electrode-tissue contact forces and a target temperature of 70° C for 30 s (50 max. output) [10,11]. A thermostat maintained a moderate flow. Figures 1 and 2 illustrate the results. With increasing contact force between tissue and electrode the electrode tip temperature increased and the average power decreased (Figure 1). Figure 2 illustrates the behavior of applied power and lesion depth. With increasing contact force the lesion depth increased until a plateau was reached. Further increase in contact force decreased lesion depth (although tip temperature was further increasing).

It is the amount of RF delivered effectively into the tissue that determines tissue heating and thus lesion generation. With increasing electrode-tissue contact a higher amount of RF power can be effectively brought into the tissue resulting in increasing lesion depth. At a certain *moderate* contact force further increase in contact force results in progressively smaller lesions because less amount of RF power is required to reach target temperature. Between 0.2 and 0.9 N (which is likely to be the bandwith in the clinical environment) the lesion depth is not much affected by the contact force and increasing contact force is balanced by decreasing applied power. These experiments were performed under stable flow conditions and in conclusion it seems likely that the flow around the electrode is of greater impact on lesion size than that of the electrode-tissue contact.

### Impact of Irrigation

Irrigation has been introduced to avoid overheating at the tissue-electrode interface, thus allowing the delivery of higher amounts of RF power for a longer duration to create relatively large lesions.

### Irrigated Power controlled RF delivery

Skrumeda et al. compared lesions created with a standard 4-mm tip catheter (RF Marinr) with those created with an irrigated tip catheter (RF Sprinklr) in animal experiments [12]. Three ablation protocols were conducted in canine left ventricles. Protocol I: standard ablation was performed in temperature controlled mode at 70° C and 90° C (120 sec). Protocol II: irrigated ablation was conducted with 30 and 50 W (30 and 120 sec). Protocol III: irrigated ablation was performed at 20 W with very long RF durations (5 and 10 min). The maximal available RF power output was 50 W. The results are given in Table 10.

With a standard electrode, lesions were larger with a target temperature of 90° C as compared to those created with a target temperature of 70° C, however, a coagulum was observed in 95% of applications with a target temperature of 90° C. The largest irrigated lesions were formed using 50 W (986±357 mm3) but were associated with craters in 54% and coagulum in 27% of the applications, respectively. Large lesions without craters and coagulum were created with irrigation using 20 W for 10 minutes (602±175 mm3). Skrumeda et al. concluded that irrigated ablation created larger lesions than standard ablation and that large lesions may be created without craters using moderate power and long duration !

### Irrigated Temperature Controlled RF delivery

Petersen et al. compared lesions produced by standard temperature controlled RF delivery (TC) with those produced by either power controlled RF delivery (PC) with a high irrigation flow rate (20 ml/min) or temperature controlled RF delivery with a low irrigation flow rate (1 ml/min) [16]. The results are given in Table 11.

Petersen et al. demonstrated that lesion size and tissue temperatures were significantly higher during irrigated tip ablation compared to standard temperature controlled RF delivery (p<0.05). Lesion volume correlated positively with tissue temperature (r=0.87). The maximum recorded tissue temperature was always 1 mm from the ablation electrode. Crater formation only occurred at tissue temperatures of greater than 100° C.

Based on the results of this in vitro study it may be concluded that irrigated temperature controlled RF delivery yields relatively large lesions without crater formation if a moderate target temperature between 60 and 70° C and a low irrigation flow rate of 1ml/min are chosen. A target temperature of greater than 70° C may result in tissue overheating and crater formation.

### Impact of irrigation flow rate

Weiss et al. investigated the influence of different flow rates on lesions produced on the thigh muscle in six sheep [17]. A total of 43 lesions was created with an irrigated tip catheter (RF Sprinklr) in a power controlled mode with 30 W target power for 30 s. A constant contact pressure of 0.1 N was maintained with a perpendicular orientation to the tissue. The results are given in Table 12.

The tissue temperatures at 7-mm depth, the lesion depth and width were not significantly different between the 3 different flow rates. The diameter measured at the surface was significantly smaller following RF applications with an irrigation flow rate of 20 ml/min due to increased cooling at the surface, which resulted also in lower tissue temperatures at a depth of 3.5 mm. Neither audible pops nor thrombus formation was observed in all applications. Based on these results a flow rate of 10 ml/min may be recommended when operating an irrigated catheter in the power controlled mode with a target power of about 30 W. The application of more than 30 W may require a higher flow rate to avoid excessive heat development at the superficial tissue layers.

### Irrigation during Atrial Flutter Ablation

Jais et al. published the results of a prospective randomized comparison of irrigated tip versus conventional tip catheters for ablation of atrial flutter [13].

Cavotricuspid ablation was performed with a conventional (n=26) or an irrigated tip catheter (n=24). RF was applied for 60 seconds with a temperature-controlled mode: 65°C to 70°C up to 70 W with a conventional catheter or 50°C up to 50 W (with a 17 ml/min saline flow rate) with the irrigated tip catheter. Complete bidirectional isthmus block was achieved for all patients. Four patients crossed over from conventional to irrigated tip catheters. The number of applications, procedure duration, and x-ray exposure were significantly higher with the conventional than with the irrigated tip catheter: 13±10 versus 5±3 pulses, 53±41 versus 27±16 minutes, and 18±14 versus 9±6 minutes, respectively. No significant side effects occurred, and the coronary angiograms of the first 30 patients after ablation was unchanged.

Jais et al. concluded that irrigated tip catheters were found to be more effective than and as safe as conventional catheters for flutter ablation, facilitating the rapid achievement of bidirectional isthmus block.

### Irrigation for difficult Accessory Pathways

Yamane et al. used an irrigated tip catheter for the ablation of accessory pathways resistant to conventional catheter ablation [14].

Among 314 accessory pathways in 301 consecutive patients, conventional ablation failed to eliminate accessory pathway conduction in 18 accessory pathways in 18 patients (5.7%), 6 of which were located in the left free wall, 5 in the middle/posterior-septal space, and 7 inside the coronary sinus (CS) or its tributaries. Irrigated tip catheter ablation was subsequently performed with temperature control mode (target temperature, 50°C), a moderate saline flow rate (17 ml/min), and a power limit of 50 W (outside CS) or 20 to 30 W (inside CS) at previously resistant sites. Seventeen of the 18 resistant accessory pathways (94%) were successfully ablated with a median of 3 applications using irrigated tip catheters. A significant increase in power delivery was achieved (20.3±11.5 versus 36.5±8.2 W; P<0.01) with irrigated tip catheters, irrespective of the accessory pathway location, particularly inside the CS or its tributaries. No serious complications occurred.

Yamane et al. concluded that irrigated tip catheter ablation is safe and effective in eliminating accessory pathway conduction resistant to conventional catheters, irrespective of the location.

### Irrigation for Ventricular Tachycardia (VT)

Nabar et al. used irrigated tip catheters for ablation of ventricular tachycardias that were resistant to conventional catheter ablation [15].

Eight patients (6 men, age 59±12 years) in whom the clinical target VT (cycle length 430±97 msec) could not be ablated using a conventional 4-mm tip RF ablation catheter underwent additional attempts to ablate this VT using an irrigated tip catheter. Ablation of the clinical target VT using an irrigated tip catheter was attempted from the left ventricle in 6 (septal, posterobasal, and inferior: 2 each) and from the right ventricle in 2 patients (both septal), by entrainment, activation, or pace mapping. A mean of 6±5 (range 2 to 15) pulses was delivered. Target VT ablation was successful in 5 patients (63%). After successful ablation, at a mean follow-up of 6.5±4 months and while taking antiarrhythmic drugs, all 5 patients were free of VT recurrences. Nabar et al. concluded that the clinical target VT could be ablated using an irrigated tip catheter in 5 (63%) of the 8 patients in whom ablation using a conventional RF catheter was unsuccessful.

## Recommendations

### 4-mm Tip Catheters

The target temperature for 4-mm tip catheters should be less than 80° C. Since tissue temperature can be markedly higher than tip temperature a higher target temperature may increase the incidence of tissue overheating associated with crater formation and coagulum formation. The lesion size is poorly correlated to tip temperature in the in vivo situation. In high flow areas the tip is cooled and more RF power is delivered to the tissue to reach target temperature resulting in relatively large lesions and vice versa. Consequently, in high flow areas in the heart the difference between tip temperature and tissue temperature is large and a lower target temperature should be considered (e.g., 60° C) whereas in low flow areas the tissue temperature is much better reflected by the tip temperature and a higher target temperature could be considered (e.g., 80° C).

The duration could be limited to 30 seconds for non-irrigated 4-mm tip electrodes. The lesion is formed within the first 30 seconds predominantly. A longer duration does not create larger lesions.

### 8-mm Tip Catheters

A larger portion of 8-mm tip catheters is exposed to the blood and thus cooled by the blood flow and a relatively large difference between tip temperature and tissue temperature can be expected. Consequently, a moderate target temperature (e.g., 60° C) should be chosen and the RF power may be limited to 50-60W to avoid tissue overheating and coagulum formation.

### Irrigated Tip Catheters: Irrigation Flow rate

An irrigation flow rate of 10ml/min may be selected in a power controlled mode with a delivered power of up to 30 W. The irrigation flow rate should be increased to 15-20 ml/min when more than 30 W are delivered to avoid excessive heat development at the superficial tissue layers.

### RF Power and RF duration

The RF duration in power controlled mode with irrigated tip catheters should be considered to be longer than 30 s. Instead of increasing the power to achieve the desired effect (which increases the likelihood of crater formation) the duration could be increased. Skrumeda demonstrated lesions of similar size with 20 W for 300 s as with 50 W for 30 s. Consequently, a moderate power of 20-35W with relatively long RF duration of 60-300 seconds should be considered to achieve relatively large lesions with a limited risk of crater formation.

## Figures and Tables

**Table 1:**
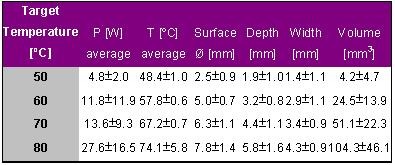
Ablation and lesion data for different target temperatures of 50, 60, 70 and 80° C. Radiofrequency energy was delivered for 60 s with a maximal available power of 100 W. Each setting was repeated 6 times and the average values are given with the corresponding standard deviation.

**Table 2:**
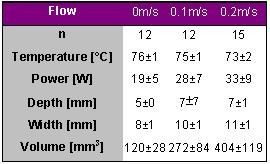
Ablation and lesion data for different flow rates of 0.0, 0.1 and 0.2 m/s. Radiofrequency energy was delivered for 60 s with a target temperature of 80° C and a maximal power of 75 W.

**Table 3:**
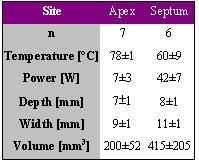
Ablation and lesion data for 2 different ablation sites, an apical site with presumably low flow and a septal site with presumably high flow. Radiofrequency energy was delivered for 60 s with a target temperature of 80° C and a maximal power of 50 W.

**Table 4:**
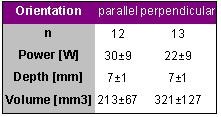
Ablation and lesion data for 2 different catheter orientations. Radiofrequency energy was delivered for 60 s with a target temperature of 80° C.

**Table 5:**
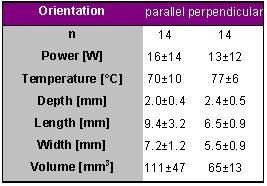
Ablation and lesion data for 2 different catheter orientations. Radiofrequency energy was delivered in vivo in the right atrium for 60 s with a target temperature of 75° C

**Table 6:**
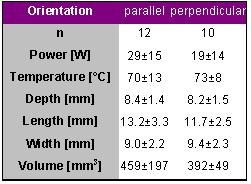
Ablation and lesion data for 2 different catheter orientations. Radiofrequency energy was delivered in vivo in the left ventricle for 60 s with a target temperature of 70° C

**Table 7:**
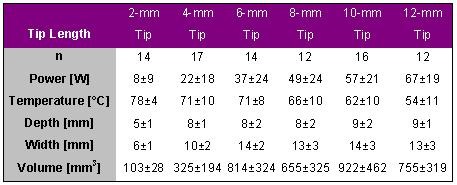
Ablation and lesion data for applications with different catheter tip lengths of 2, 4, 6, 8, 10 and 12-mm. Radiofrequency energy was delivered in vivo in the left ventricle for 60 s with a target temperature of 80° C and a maximal power of 75 W.

**Table 8:**
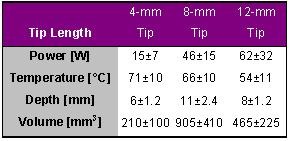
Ablation and lesion data for applications with different catheter tip lengths of 4, 8 and 12-mm. Radiofrequency energy was delivered in vivo in the left ventricle for 60 s with a target temperature of 80° C and a maximal power of 100 W.

**Table 9:**
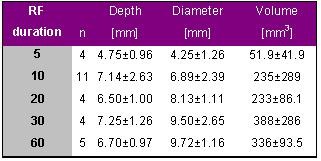
Lesion data for applications with different durations of radiofrequency delivery of 5, 10, 20, 30 and 60 s. Radiofrequency energy was delivered in vivo at ventricular sites with a constant power of 25 W.

**Table 10:**
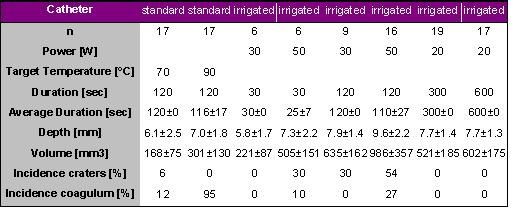
Ablation and lesion data for applications with standard tip electrodes and irrigated tip electrodes. The results for different target temperatures, power settings and durations of radiofrequency delivery and the incidence of craters and coagulum formation are given.

**Table 11:**
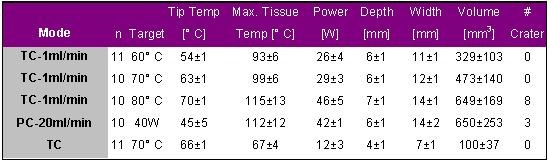
Ablation and lesion data for applications with standard tip electrodes either in temperature controlled or power controlled mode and irrigated tip electrodes with a low irrigation flow rate of 1ml/min and different target temperatures.

**Table 12:**
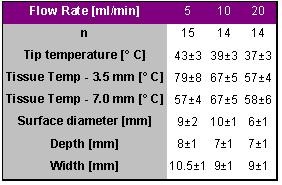
Ablation and lesion data for applications with irrigated tip electrodes with different irrigation flow rates of 5, 10 and 20 ml/min. Besides the catheter tip temperature, the temperature at a depth of 3.5 and 7.0 mm is given.`

## References

[R1] Erez A, Shitzer A (1980). Controlled destruction and temperature distributions in biological tissues subjected to monoactive electrocoagulation. J Biomech Eng.

[R2] Petersen HH, Chen X, Pietersen A (1999). Lesion Dimmensions During Temperature-Controlled Radiofrequency Catheter Ablation of Left Ventricular Porcine Myocardium. Circulation.

[R3] Petersen HH, Chen X, Pietersen A (1998). Lesion size in relation to ablation site during radiofrequency ablation. Pacing Clin Electrophysiol.

[R4] Petersen HH, Chen X, Pietersen A (1999). Temperature-controlled radiofrequency ablation of cardiac tissue: an in vitro study of the impact of electrode orientation, electrode tissue contact pressure and external convective cooling. J Interv Card Electrophysiol.

[R5] Chan RC, Johnson SB, Seward JB (2002). The effect of ablation electrode length and catheter tip to endocardial orientation on radiofrequency lesion size in the canine right atrium. Pacing Clin Electrophysiol.

[R6] Chugh SS, Chan RC, Johnson SB (1999). Catheter tip orientation affects radiofrequency ablation lesion size in the canine left ventricle. Pacing Clin Electrophysiol.

[R7] Langberg JJ, Gallagher M, Strickberger SA (1993). Temperature-guided radiofrequency catheter ablation with very large distal electrodes. Circulation.

[R8] Tsai CF, Tai CT, Yu WC (1999). Is 8-mm more effective than 4-mm tip electrode catheter for ablation of typical atrial flutter?. Circulation.

[R9] Simmers TA, Wittkampf FH, Hauer RN (1994). In vivo ventricular lesion growth in radiofrequency catheter ablation. Pacing Clin Electrophysiol.

[R10] Eick OJ (1999). Der Elektrodenkontakt am Herzgewebe waehrend einer Hochfrequenzablation: Klinische Bedeutung und Entwicklung einer Messmethode.

[R11] Eick OJ, Wittkampf FH, Bronneberg T (1998). The LETR-Principle: a novel method to assess electrode-tissue contact in radiofrequency ablation. J Cardiovasc Electrophysiol.

[R12] Skrumeda LL, Mehra R (1998). Comparison of standard and irrigated radiofrequency ablation in the canine ventricle. J Cardiovasc Electrophysiol.

[R13] Jais P, Shah DC, Haissaguerre M (2000). Prospective randomized comparison of irrigated-tip versus conventional-tip catheters for ablation of common flutter. Circulation.

[R14] Yamane T, Jais P, Shah DC (2000). Efficacy and safety of an irrigated-tip catheter for the ablation of accessory pathways resistant to conventional radiofrequency ablation. Circulation.

[R15] Nabar A, Rodriguez LM, Timmermans C (2001). Use of a saline-irrigated tip catheter for ablation of ventricular tachycardia resistant to conventional radiofrequency ablation: early experience. J Cardiovasc Electrophysiol.

[R16] Petersen HH, Chen X, Pietersen A (2000). Tissue temperatures and lesion size during irrigated tip catheter radiofrequency ablation: an in vitro comparison of temperature-controlled irrigated tip ablation, power-controlled irrigated tip ablation, and standard temperature-controlled ablation. Pacing Clin. Electrophysiol.

[R17] Weiss C, Antz M, Eick O (2002). Radiofrequency catheter ablation using cooled electrodes: impact of irrigation flow rate and catheter contact pressure on lesion dimensions. Pacing Clin.Electrophysiol.

